# Patients’ Preference for Participation in Medical Decision-Making: *Secondary Analysis of the BEDSIDE-OUTSIDE Trial*

**DOI:** 10.1007/s11606-022-07775-z

**Published:** 2022-09-09

**Authors:** Christoph Becker, Sebastian Gross, Martina Gamp, Katharina Beck, Simon A. Amacher, Jonas Mueller, Chantal Bohren, René Blatter, Rainer Schaefert, Philipp Schuetz, Joerg Leuppi, Stefano Bassetti, Sabina Hunziker

**Affiliations:** 1 Medical Communication, Department of Psychosomatic Medicine, University Hospital Basel, Petersgraben 4, 4031 Basel, CH Switzerland; 2 Emergency Department, University Hospital Basel, Basel, Switzerland; 3 Intensive Care Unit, University Hospital Basel, Basel, Switzerland; 4 Faculty of Medicine, University of Basel, Basel, Switzerland; 5 Division of Internal Medicine, Kantonsspital Aarau, Aarau, Switzerland; 6 Division of Internal Medicine, Kantonsspital Baselland, Liestal, Switzerland; 7 Division of Internal Medicine, University Hospital Basel, Basel, Switzerland

**Keywords:** decisional control preference, decision-making, hospital medicine, satisfaction, quality of care

## Abstract

**Background:**

Patients may prefer different levels of involvement in decision-making regarding their medical care which may influence their medical knowledge.

**Objective:**

We investigated associations of patients’ decisional control preference (DCP) with their medical knowledge, ward round performance measures (e.g., duration, occurrence of sensitive topics), and perceived quality of care measures (e.g., trust in the healthcare team, satisfaction with hospital stay).

**Design:**

This is a secondary analysis of a randomized controlled multicenter trial conducted between 2017 and 2019 at 3 Swiss teaching hospitals.

**Participants:**

Adult patients that were hospitalized for inpatient care.

**Main Measures:**

The primary outcome was patients’ subjective average knowledge of their medical care (rated on a visual analog scale from 0 to 100). We classified patients as active, collaborative, and passive according to the Control Preference Scale. Data collection was performed before, during, and after the ward round.

**Key Results:**

Among the 761 included patients, those with a passive DCP had a similar subjective average (mean ± SD) knowledge (81.3 ± 19.4 points) compared to patients with a collaborative DCP (78.7 ± 20.3 points) and active DCP (81.3 ± 21.5 points), *p* = 0.25. Regarding patients’ trust in physicians and nurses, we found that patients with an active vs. passive DCP reported significantly less trust in physicians (adjusted difference, − 5.08 [95% CI, − 8.69 to − 1.48 points], *p* = 0.006) and in nurses (adjusted difference, − 3.41 [95% CI, − 6.51 to − 0.31 points], *p* = 0.031). Also, patients with an active vs. passive DCP were significantly less satisfied with their hospital stay (adjusted difference, − 7.17 [95% CI, − 11.01 to − 3.34 points], *p* < 0.001).

**Conclusion:**

Patients with active DCP have lower trust in the healthcare team and lower overall satisfaction despite similar perceived medical knowledge. The knowledge of a patient’s DCP may help to individualize patient-centered care. A personalized approach may improve the patient-physician relationship and increase patients’ satisfaction with medical care.

**Trial Registration:**

ClinicalTrials.gov (NCT03210987).

**Supplementary Information:**

The online version contains supplementary material available at 10.1007/s11606-022-07775-z.

## INTRODUCTION

During hospitalization, decision-making regarding patient management and further steps regularly occurs during ward rounds.^1^ Ward rounds not only provide the opportunity to inform patients regarding their current condition and treatment but also ensure that these treatments are in alignment with patients’ preferences and needs.^2,3^ Patients’ involvement in decision-making contributes to a better quality of care and is therefore an important priority during ward rounds.^3–7^

However, there may be differences among patients regarding their preferences in active involvement. In fact, some patients may differ regarding their preferred role in medical decision-making, a construct which is described as decisional control preference (DCP).^8^ Several years ago, Degner et al. introduced the control preference scale, an instrument to assess the degree of control an individual patient prefers during healthcare decision-making.^9^ Especially in the context of decision-making in oncology and palliative care, knowledge about a patient’s DCPs has become an important focus of care in the recent years and may help to individualize treatment according to patients’ preferences. However, in clinical practice, a patient’s DCP is often unknown and physicians may fail to actively involve those who would prefer participation in decision-making and vice versa.^10^ Moreover, Hamann et al. suggested that DCPs vary not only between patients but also across different diseases and circumstances.^8^

Degner distinguished between patients that indicate that they prefer to leave decisions up to the healthcare specialist (passive DCP), patients that prefer to choose between treatment options independently (active DCP), and patients that wish to make a shared decision with their healthcare specialists (collaborative DCP).^9^ Research has found passive DCP to be associated with low education, older age, and ethnic minority.^11^ In contrast, patients with active or collaborative DCP are often younger, have a higher education, and demand more detailed medical information to participate in the decision process.^11–13^ Also, previous studies suggest that patients’ preferences may influence patient-related experience measures.^14,15^

Until today, most of the studies on DCP have focused on well-defined and homogenous patient populations such as patients with cancer or end-of life settings. Research in general medical populations is scarce. Previous literature suggest that physicians may provide passive patients with fewer medical information.^16^ Thus, these patients may be passed over and as a result have less knowledge regarding their illness.

Therefore, we investigated the association between patients’ DCP, their medical knowledge, and different aspects of perceived quality of care among patients hospitalized on general medical wards. Patients had been recruited in a prior multicenter randomized controlled trial (RCT), the BEDSIDE-OUTSIDE Trial.^17^

## METHODS

### Study Setting and Population

This is a secondary analysis of the BEDSIDE-OUTSIDE Study, a multicenter RCT with the aim to assess the effects of bedside case presentation during ward rounds on patients’ medical knowledge. The design, statistical analysis, and primary results have been published elsewhere.^17^

In brief, patients hospitalized in the medical divisions of three Swiss tertiary care hospitals (University Hospital Basel, Kantonsspital Aarau, and Kantonsspital Baselland) between July 2017 and October 2019 were eligible to participate in the trial.

Consecutive adult patients newly admitted to medical wards for inpatient care who had their first weekly consultant ward round during hospitalization were recruited and randomized to the “bedside case-presentation group” or the “outside-the-room case-presentation group” in a 1:1 ratio.

During a consultant ward round, the team, which oversees the patients’ treatment (attending physician, resident, nurse, pharmacist, and medical students), is accompanied by a consultant, e.g., the head of service or one of their deputies. As the consultant does not know the patient in advance, a resident presents the patient to the healthcare team. Afterwards, the team discusses the patient’s case in more detail considering patient history and current laboratory findings as well as potential diagnostic and therapeutic approaches.

Patients with cognitive impairment (e.g., dementia/delirium) or severe hearing impairment and patients who did not have the proficiency of the local language(s) (German or French) or who had previously participated in the study were excluded. All patients were aware about the study purpose and provided written informed consent.

The study was approved by the local Ethics Committee (Northwest and Central Switzerland, EKNZ, 2017-00991) and registered at clinicaltrials.gov (NCT03210987).

This study adheres to the CONSORT guidelines.^18^

### Data Collection

Data collection occurred at different timepoints: one day before the ward round, during the ward round, and in the afternoon after the ward round.

One day before the ward round, a member of the study team gathered baseline patient data including socio-demographics, patients’ conditions leading to hospitalization, ongoing medical investigations, and therapeutic treatments as well as patients’ comorbidities extracted from the electronic patient chart. Also, we calculated the Charlson Comorbidity Index^19^ based on these data. Further, we assessed patients’ perceived health status using the three-level version of EuroQol-5-Dimension questionnaire (EQ-5D-3L),^20^ which includes five dimensions covering mobility, self-care, daily-life activities, and pain as well as anxiety or depression. Finally, we assessed patients’ preferences for participation in clinical decision-making through the Control Preference Scale^21^ before the ward round. The Control Preference Scale asks patients how much control in decision-making regarding their own care they would like to take. Patients’ responses can be allocated to five categories: active, active-shared, collaborative, passive-shared, and passive control preference. We collapsed these options into the categories active (combining active and active-shared), collaborative, and passive (combining passive-shared and passive) control preference, as suggested in previous research.^10^

A member of the research team joined the ward round to audiotape the ward round on an iPad (Apple®). The audiotapes were later analyzed for the duration of the ward round for each patient and the occurrence of sensitive topics.

After the ward round, a different blinded member of the research team interviewed patients through a face-to-face interview with a structured questionnaire to assess patients’ subjective average knowledge of their medical care (primary endpoint) as well as patients’ perception regarding participation during ward round (e.g., patients’ estimation of participation during ward round), patients’ discomfort during ward round (e.g., confusion due to medical terms used during ward round), and patients’ perception regarding quality of care (e.g., trust in the healthcare team, satisfaction with hospital stay).

All data were directly entered into an electronic data file (SecuTrial®)

### Outcome Measures

#### Primary Endpoint

In line with the main study, the primary endpoint of our secondary analysis was patients’ average subjective knowledge regarding their medical care across the different dimensions “understanding of their disease” and “understanding of the therapeutic approach” as well as “understanding of further plans of care.” All dimensions were rated by the patient on a visual analog scale from 0 to 100 (0 “I have no knowledge about the situation” to 100 “I have the best possible knowledge about the situation”).

#### Secondary Endpoints

In accordance with the primary endpoint, we rated patients’ objective knowledge within the three dimensions, i.e., understanding of the disease, therapeutic approach, and further plans of care. After the ward rounds, we asked patients to recall the current main diagnoses, therapeutic measures, and further plans of care. A blinded study member then compared the responses with the medical information from the medical chart and rated them on a predefined scale from 0 to 100.

We also assessed different aspects of perceived quality of care such as satisfaction with hospital stay or patients’ trust in the healthcare team, all rated on a visual analog scale from 0 to 100.

Finally, we analyzed the audio recordings to assess timeliness of the ward rounds per patient and whether sensitive topics (e.g., nonadherence, treatment failure, social issues) were discussed on a nominal 3-point scale (1 = yes, 2 = no, 3 = not applicable).

More detailed information regarding definition and assessments of primary and secondary outcomes are given in the supplement.

### Statistical Analysis

We used descriptive statistics such as frequencies as well as means and standard deviations to describe characteristics of the study population. To compare primary and secondary outcomes between patients with a passive, collaborative, and active DCP, we performed one-way ANOVA.

Additionally, to evaluate differences between patients with passive and collaborative as well as passive and active DCP regarding primary and secondary outcomes, we conducted linear and logistic regression analyses. Further, we calculated linear and logistic regression models adjusted for study center and randomization. In an additional exploratory analysis, we also adjusted the model for age, gender, and education (presented in the supplement).

Finally, we performed subgroup analyses for the different DCP categories stratified by study intervention for patients’ subjective knowledge and occurrence of sensitive topics as well as duration of ward round and calculated interaction terms. All analyses were performed using the intention-to-treat analysis sample of the original trial. A *p* value of < 0.05 (two-tailed) was considered statistically significant. We used STATA 15.0 (Stata Corp., College Station, TX, USA) for all statistical analyses.

## RESULTS

A total of 919 patients were enrolled in the original trial (see eFigure 1). Of these patients, 158 had missing information regarding patients’ DCP and were excluded from this analysis.

Baseline characteristics of the remaining 761 patients, stratified among the 3 groups of patients’ control preference, are shown in Table 1. Overall, 62.2% of patients had a collaborative DCP, and 22.4% preferred a passive and 15.4% an active role, regarding medical decision-making. Patients were on average 64.5 years old and 39% were female.

**Table 1:** Characteristics of Patients at Trial Entry Stratified for Intervention Arms

	*n*	All	DCP = passive	DCP = SDM	DCP = active	*p*
		*n* = 761	*n* = 171	*n* = 473	*n* = 117	
**Socio-demographic factors**						
***Age, years (mean, SD)***	760	64.4 (15.8)	65.3 (15.9)	64.6 (15.4)	62.5 (17.0)	0.31
***Age categories, years (n, %)***	760					
18–25		22 (2.9%)	6 (3.5%)	11 (2.3%)	5 (4.3%)	0.30
26–50		110 (14.5%)	23 (13.5%)	64 (13.6%)	23 (19.7%)	
51–75		426 (56.1%)	90 (52.6%)	278 (58.9%)	58 (49.6%)	
76–95		202 (26.6%)	52 (30.4%)	119 (25.2%)	31 (26.5%)	
***Female gender (n, %)***	755	294 (38.9%)	55 (32.2%)	193 (40.8%)	48 (41.0%)	0.12
***Number of children (mean, SD)***	753	2.0 (6.3)	2.8 (10.6)	2.5 (9.0)	1.4 (1.4)	0.42
***Citizenship***	754					
Switzerland		649 (86.1%)	146 (85.4%)	409 (86.5%)	99 (84.6%)	0.009
Germany		48 (6.4%)	9 (5.3%)	25 (5.3%)	15 (12.8%)	
Other		57 (7.6%)	16 (9.4%)	39 (8.2%)	3 (2.6%)	
***Highest level of education***	760					
High school		131 (17.2%)	30 (17.5%)	83 (17.5%)	18 (16.2%)	0.95
Apprenticeship		534 (70.2%)	119 (69.6%)	334 (70.6%)	81 (69.2%)	
College/university		95 (12.5%)	22 (12.9%)	56 (11.8%)	17 (14.5%)	
**Health-related factors**						
***Main admission diagnosis (n, %)***	761					
Coronary heart disease		77 (10.1%)	24 (14.0%)	41 (8.7%)	12 (10.3%)	0.27
Congestive heart failure		67 (8.8%)	23 (13.5%)	37 (7.8%)	7 (6.0%)	
Other cardiovascular diseases		79 (10.4%)	13 (7.6%)	55 (11.6%)	11 (9.4%)	
Infections		175 (23.0%)	39 (22.8%)	105 (22.2%)	31 (26.5%)	
Gastro-intestinal diseases		51 (6.7%)	11 (6.4%)	31 (6.6%)	9 (7.7%)	
Metabolism		42 (5.5%)	12 (7.0%)	25 (5.3%)	5 (4.3%)	
Malignant neoplasm		63 (8.3%)	13 (7.6%)	41 (8.7%)	9 (7.7%)	
Other		207 (27.2%)	36 (21.1%)	138 (29.2%)	33 (28.2%)	
***Comorbidities***						
*Charlson Comorbidity Index (mean, SD)*	761	4.38 (2.90)	4.19 (2.65)	4.42 (2.85)	4.45 (3.44)	0.64
***Quality of life (Euroqol)***						
EQ-5D	744	57.1 (22.6)	58.2 (21.5)	56.4 (22.9)	58.0 (23.0)	0.63
EQ-VAS	721	0.7 (0.3)	0.8 (0.3)	0.7 (0.3)	0.7 (0.3)	0.26

To estimate quality of life, we used the EQ-5D index (values range between − 0.205 and 1, with higher values indicating better quality of life) and EQ-5D VAS (values range between 0 and 100, with higher values indicating better self-perceived health status)

*DCP* decisional control preference, *SD* standard deviation, *EQ-5D* European Quality of Life 5 Dimensions, *VAS* visual analog scale

### Primary Endpoint: Patients’ Subjective Knowledge

Patients with a passive DCP reported a similar subjective knowledge (points) (mean, 81.3 ± 19.4) compared to patients with a collaborative DCP (mean, 78.7 ± 20.3) and active DCP (mean, 81.3 ± 21.5), *p* = 0.25 (Table 2 and supplement). There was no significant difference between passive DCP and collaborative DCP (adjusted difference, − 2.52 [95% CI, − 6.06 to 1.03 points]; *p* = 0.164) as well as between passive DCP and active DCP (adjusted difference, − 0.04 points [95% CI, − 4.81 to 4.73 points]; *p* = 0.986).

**Table 2:** Primary and Secondary Outcomes

Outcome measures	*n*	All	DCP = passive	DCP = SDM	DCP = active	*p*	Adjusted difference or OR (95% CI), *p* passive vs SDM Model 1*	*p*	Adjusted difference or OR (95% CI) passive vs active Model 1*	*p*
Primary endpoint, mean (SD)										
Subjective overall knowledge about their medical care (VAS 0–100)	761	79.7 (20.3)	81.3 (19.4)	78.7 (20.3)	81.3 (21.5)	0.25	− 2.52 (− 6.06, 1.03)	0.164	− 0.04 (− 4.81, 4.73)	0.986
Secondary endpoints, mean (SD)										
Objective overall knowledge about their medical care (rated by study team)	761	71.9 (24.6)	70.2 (25.4)	72.4 (24.2)	72.6 (25.0)	0.59	2.11 (− 2.2, 6.42)	0.336	2.43 (− 3.37, 8.22)	0.411
Participation during the ward round, mean (SD) or *n* (%)										
Total duration of ward round per patient (min)	761	12.86 (5.33)	12.32 (5.02)	12.69 (5.30)	13.96 (6.08)	**0.032**	0.49 (− 0.40, 1.38)	0.28	1.66 (0.46, 2.86)	**0.007**
I was encouraged to address personal topics (VAS 0–100)	550	85.4 (29.6)	85.4 (29.6)	83.5 (31.3)	93.5 (19.6)	**0.018**	− 1.89 (− 8.24, 4.46)	0.56	7.82 (− 0.52, 16.15)	0.066
Estimation of my participation during the ward round (VAS 0–100)	736	62.6 (35.5)	61.3 (31.5)	63.0 (37.5)	62.5 (33.1)	0.87	1.68 (− 4.65, 8.01)	0.603	1.38 (− 7.09, 9.86)	0.749
Time spent with physicians was sufficient (VAS 0–100)	691	89.7 (18.9)	91.4 (16.7)	89.1 (19.3)	89.9 (20.4)	0.44	− 2.28 (− 5.78, 1.22)	0.201	− 1.49 (− 6.19, 3.21)	0.534
All my questions were answered (VAS 0–100)	647	91.5 (17.9)	92.2 (17.8)	90.5 (19.0)	94.7 (12.8)	0.084	− 1.74 (− 5.16, 1.67)	0.317	2.52 (− 1.96, 6.99)	0.27
Occurrence of sensitive topic during the ward round	760	536 (70.5%)	114 (66.7%)	330 (69.8%)	92 (79.3%)	0.059	1.17 (0.8, 1.71)	0.428	1.92 (1.1, 3.37)	**0.022**
Patient perception regarding discomfort during the ward round										
Medical terms used during ward round were confusing (VAS 0–100)	735	16.7 (28.2)	15.4 (27.0)	16.7 (28.1)	18.7 (30.0)	0.63	1.23 (− 3.74, 6.19)	0.628	3.5 (− 3.22, 10.22)	0.307
I felt discomfort due to the interactions during the ward round (VAS 0–100)	753	6.0 (18.0)	4.0 (14.5)	6.3 (18.6)	7.6 (19.9)	0.21	2.44 (− 0.7, 5.59)	0.128	3.66 (− 0.56, 7.89)	0.089
Discussion within healthcare team caused upset (VAS 0–100)	651	5.6 (17.5)	5.6 (18.5)	5.4 (16.8)	6.5 (18.7)	0.87	− 0.15 (− 3.45, 3.15)	0.929	0.82 (− 3.72, 5.36)	0.723
Patients’ perception regarding quality of care (VAS 0–100)										
I felt “in good hands” in this hospital	756	90.3 (15.0)	92.2 (13.4)	90.0 (15.1)	88.9 (16.7)	0.15	− 2.23 (− 4.85, 0.39)	0.096	− 3.27 (− 6.8, 0.25)	0.069
I have trust in the physician team	751	90.7 (15.2)	93.3 (11.9)	90.4 (15.0)	88.2 (19.3)	**0.016**	− 2.95 (− 5.62, − 0.28)	**0.03**	− 5.08 (− 8.69, − 1.48)	**0.006**
I have trust in the nursing team	750	92.6 (13.1)	94.7 (9.2)	92.2 (13.3)	91.2 (16.3)	0.055	− 2.42 (− 4.72, − 0.13)	**0.039**	− 3.41 (− 6.51, − 0.31)	**0.031**
There is good collaboration of physicians and nurses	697	90.6 (14.1)	92.2 (11.6)	90.4 (14.3)	88.8 (16.2)	0.14	− 1.78 (− 4.36, 0.8)	0.175	− 3.43 (− 6.88, 0.02)	0.051
I feel physicians have high competence to treat the current illness	725	91.3 (34.4)	92.1 (15.2)	89.8 (16.4)	96.1 (78.8)	0.21	− 2.34 (− 8.47, 3.8)	0.455	4.03 (− 4.2, 12.26)	0.337
I feel nurses have high competence to treat the current illness	729	92.1 (20.7)	95.4 (34.2)	91.2 (13.9)	90.7 (16.2)	0.058	− 4.25 (− 7.94, − 0.56)	**0.024**	− 4.71 (− 9.64, 0.23)	0.062
Overall satisfaction with hospital stay	758	88.2 (16.6)	91.2 (13.4)	88.2 (16.7)	83.9 (19.5)	**0.001**	− 3.01 (− 5.86, − 0.16)	**0.038**	− 7.17 (− 11.01, − 3.34)	**< 0.001**

*DCP* decisional control preference, *OR* odds ratio, *SD* standard deviation, *CI* confidence interval, *VAS* visual analog scale

*Model 1 adjusted for study center and randomization

An additional model adjusted for age, gender, and education showed similar results (supplement).

### Secondary Endpoints

Objective knowledge (points) was similar between patients reporting a passive, collaborative, and active DCP (mean, 70.2 ± 25.4), (72.4 ± 24.2), and (72.6 ± 25) respectively, *p* = 0.59. Regression analyses showed no significant differences between passive DCP and collaborative DCP (adjusted difference, 2.11 points [95% CI, − 2.2 to 6.42 points]; *p* = 0.336) as well as between passive DCP and active DCP (adjusted difference, 2.43 points [95% CI, − 3.37 to 8.22 points]; *p* = 0.411).

There was a significant correlation between patients’ subjective and objective knowledge (Spearman’s rho, 0.22; *p* < 0.001)

Regarding trust in the physician team, the mean (± SD) did significantly differ between patients reporting a passive, collaborative, and active DCP (93.3 ± 11.9 vs. 90.4 ± 15.0 vs. 88.2 ± 19.3; *p* = 0.016). Compared to patients with passive DCP, patients with active DCP reported significantly less trust in physicians (adjusted difference, − 5.08 [95% CI, − 8.69 to − 1.48 points]; *p* = 0.006). Overall, mean (± SD) trust in the nursing team was similar between patients reporting a passive, collaborative, and active DCP (94.7 ± 9.2 vs. 92.2 ± 13.3 vs. 91.2 ± 16.3; *p* = 0.055). Compared to patients with passive DCP, patients with collaborative DCP (adjusted difference, − 2.42 points [95% CI, − 4.72 to − 0.13 points]; *p* = 0.039) and active DCP (adjusted difference, − 3.41 points [95% CI, − 6.51 to − 0.31 points]; *p* = 0.031) reported lower trust in the nursing team. Further, regarding satisfaction with hospital stay, there was a significant difference in mean (±SD) satisfaction between patients reporting a passive, collaborative, and active DCP (91.2 ± 13.4 vs. 88.2 ± 16.7 vs. 83.9 ± 19.5; *p* = 0.001). Compared to passive patients, active patients (adjusted difference, − 7.17 points [95% CI, − 11.01 to − 3.34 points]; *p* < 0.001) and collaborative patients (adjusted difference, − 3.01 points [95% CI, − 5.86 to − 0.16 points], *p* = 0.038) were significantly less satisfied with their hospital stay.

Duration of ward rounds (mean (± SD) minutes) did significantly differ between patients reporting a passive, collaborative, and active DCP (12.32 ± 5.02 vs. 12.69 ± 5.30 vs. 13.96 ± 6.08; *p* = 0.032). Duration per patient was on average 1.5 min shorter in patients reporting a passive DCP compared to active DCP (adjusted difference 1.66 min [95% CI, 0.46 to 2.86 points]; *p* = 0.007).

Sensitive topics were less often addressed in patients with passive DCP compared to patients with active DCP (OR, 1.92 [95% CI, 1.10, to 3.37], *p* = 0.022). Still, patients with passive, collaborative, and active DCP estimated their participation similarly during ward rounds. Regression analyses showed no significant differences between passive DCP and collaborative DCP (adjusted difference 1.68 points [95% CI, − 4.65 to 8.01 points]; *p* = 0.603) as well as between passive DCP and active DCP (1.38 points [95% CI, − 7.09 to 9.86 points]; *p* = 0.749).

### Subgroup Analyses According to Randomization Groups (Bedside Vs. Outside-the-Room Case Presentation)

**Figure 1:**
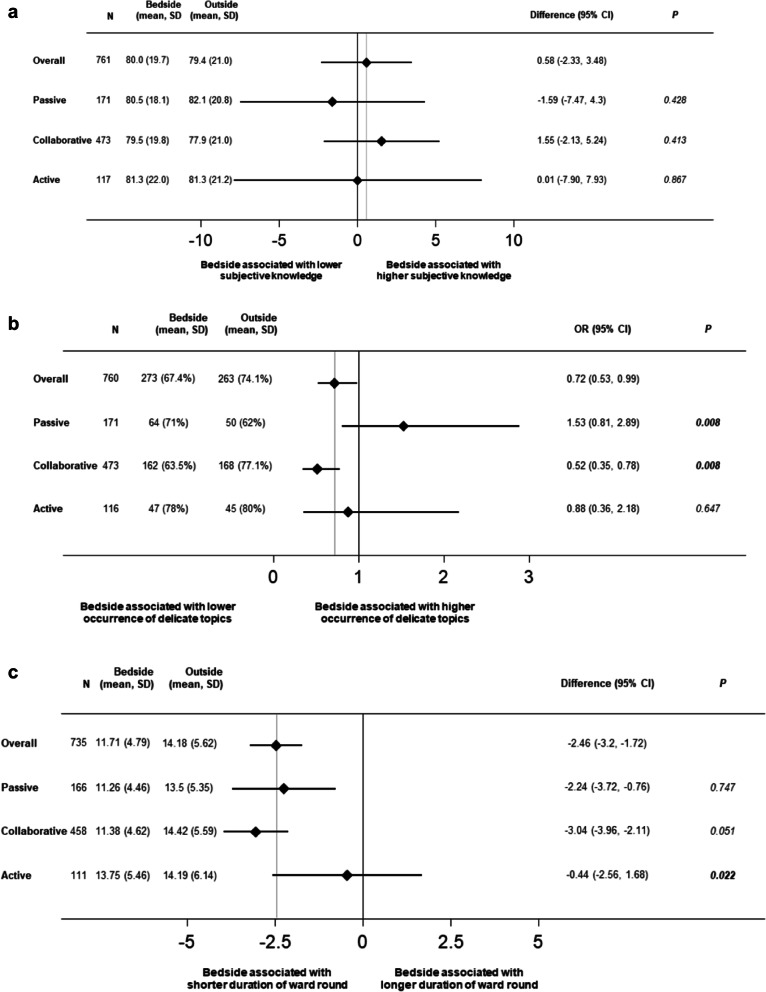
a Patients’ subjective knowledge regarding their medical care according to patients’ DCP stratified for study intervention. Legend: DCP, decisional control preference; SD, standard deviation; CI, confidence interval. b Occurrence of sensitive topics according to patients’ DCP stratified for study intervention. Legend: DCP, decisional control preference; OR, odds ratio; CI, confidence interval. c Duration of ward round according to patients’ DCP stratified for study intervention. Legend: DCP, decisional control preference; SD, standard deviation; CI, confidence interval

Regarding subjective knowledge, there were no significant differences between groups for patients with either a passive DCP, collaborative DCP, or active DCP (Table 3 and Fig. 1a–c).

**Table 3:** Primary and Secondary Outcomes Stratified by Randomization Group

Subgroups	*n*	Bedside (mean, SD)	Outside (mean, SD)	Difference or OR (95% CI)	*p*	*p* of interaction
		406	355			
Patients’ subjective overall knowledge about their medical care (VAS 0–100)						
Passive	171	80.5 (18.1)	82.1 (20.8)	− 1.59 (− 7.47, 4.3)	0.428	0.664
Collaborative	473	79.5 (19.8)	77.9 (21.0)	1.55 (− 2.13, 5.24)	0.413	
Active	117	81.3 (22.0)	81.3 (21.2)	0.01 (− 7.90, 7.93)	0.867	
Patients’ objective overall knowledge about their medical care (VAS 0–100)						
Passive	171	74.0 (24.0)	66.1 (26.5)	7.89 (0.27, 15.52)	0.077	0.262
Collaborative	473	72.3 (24.3)	72.5 (24.2)	− 0.23 (− 4.62, 4.16)	0.106	
Active	117	73.9 (24.4)	71.3 (25.9)	2.60 (− 6.60, 11.81)	0.896	
Occurrence of sensitive topics (*n*, %)						
Passive (*n* = 90)	171	64 (71%)	50 (62%)	1.53 (0.81, 2.89)	**0.008**	0.106
Collaborative	473	162 (63.5%)	168 (77.1%)	0.52 (0.35, 0.78)	**0.008**	
Active	116	47 (78%)	45 (80%)	0.88 (0.36, 2.18)	0.647	
Duration of ward round (min)						
Passive	166	11.26 (4.46)	13.5 (5.35)	− 2.24 (− 3.72, − 0.76)	0.747	0.256
Collaborative	458	11.38 (4.62)	14.42 (5.59)	− 3.04 (− 3.96, − 2.11)	0.051	
Active	111	13.75 (5.46)	14.19 (6.14)	− 0.44 (− 2.56, 1.68)	**0.022**	
Overall satisfaction with hospital stay						
Passive	171	90.1 (13.7)	92.4 (13.0)	− 2.25 (− 6.3, 1.8)	0.307	0.09
Collaborative	470	88.0 (17.2)	88.4 (16.2)	− 0.38 (− 3.42, 2.67)	0.683	
Active	117	86.2 (19.9)	81.4 (18.9)	4.78 (− 2.33, 11.9)	0.088	
I have trust in the physician team, mean (SD)						
Passive	169	92.3 (12.8)	94.4 (10.6)	− 2.1 (− 5.7, 1.51)	0.281	0.14
Collaborative	468	90.4 (15.1)	90.3 (14.9)	0.06 (− 2.68, 2.8)	0.932	
Active	114	89.9 (17.8)	86.5 (20.8)	3.43 (− 3.75, 10.61)	0.199	
I have trust in the nursing team, mean (SD)						
Passive	170	94.2 (9.3)	95.2 (9.1)	− 1.01 (− 3.8, 1.78)	0.719	0.544
Collaborative	466	92.0 (14.2)	92.5 (12.2)	− 0.5 (− 2.94, 1.93)	0.857	
Active	114	91.7 (17.7)	90.7 (14.8)	1.04 (− 5.05, 7.12)	0.526	
I feel physicians have high competence to treat the current illness, mean (SD)						
Passive	166	91.8 (17.4)	92.4 (12.3)	− 0.56 (− 5.24, 4.11)	0.672	0.787
Collaborative	446	89.4 (17.6)	90.4 (14.8)	− 1.02 (− 4.09, 2.05)	0.212	
Active	113	89.3 (19.7)	88.5 (19.6)	3.70 (− 6.54, 8.14)	0.609	
I feel nurses have high competence to treat the current illness, mean (SD)						
Passive	166	92.2 (14.1)	99.1 (47.5)	− 6.91 (− 17.4, 3.58)	0.055	0.076
Collaborative	449	91.1 (14.3)	91.3 (13.4)	− 0.25 (− 2.84, 2.34)	0.319	
Active	114	91.5 (17.5)	90.0 (14.9)	1.47 (− 4.57, 7.51)	0.401	

*SD* standard deviation, *OR* odds ratio, *CI* confidence interval

In patients with a collaborative DCP, sensitive topics were significantly less often addressed during bedside compared to outside-the-room case presentation (63.5% vs. 77.1%; OR 0.52 [95% CI, 0.35 to 0.78 points], *p* = 0.008). However, in patients with a passive DCP, sensitive topics occurred more frequently if the case presentation was conducted at the bedside (71% vs. 62%; OR 1.53 [95% CI, 0.81 to 2.89 points], *p* = 0.008, *p* of interaction = 0.106).

While bedside case presentation resulted in shorter ward rounds in patients with a passive and collaborative DCP, durations of ward rounds in patients with active DCP were similar between bedside and outside-the-room case presentation.

## DISCUSSION

Results of this secondary analysis of a large multicenter trial investigating differences in medical knowledge and perceived quality of care during hospitalization in patients according to their DCP are threefold. First, we found that most patients (i.e., 3 in 4 patients) prefer to participate in medical decision-making actively or collaboratively and only a minority has a passive DCP. Second, DCP is not associated with patients’ subjective and objective knowledge regarding their medical care. Third, although knowledge was similar between groups, patients with an active DCP were significantly more critical regarding their medical care with lower trust in the healthcare team and lower satisfaction with their overall hospital stay. Several points of this ancillary project are worth mentioning.

Results of our main trial indicated that patients have similar subjective and objective knowledge regardless of patient case presentations being conducted at the bedside or outside the room.^22^ However, it remained unclear whether this finding is true for all patients, or whether there are differences according to a patient’s DCP. This ancillary analysis now confirms that DCP has little influence on patients’ knowledge and may not be used as an indicator regarding best place for conducting ward rounds.

While DCP has been investigated in different specific patient populations (e.g., patients with cancer), there has been little evidence in unselected medical inpatients. Herein, our analysis provides important new insights in a large sample of patients with different main diagnoses. There are few studies looking specifically at associations between patients’ control preference and their medical knowledge. An older study from Germany found a weak correlation between preferences and knowledge in a cohort of patients with multiple sclerosis (MS).^23^ Patients with an active DCP had a slightly higher knowledge regarding their disease and medication. However, in the MS cohort, approximately 40% of patients showed an active DCP compared to 15.4% in our trial. Since patients may prefer more involvement in scenarios with chronic conditions or quality-of-life issues,^24^ the results may not be applicable to a broader population. Researchers have concluded that patient knowledge facilitates participation in the decision-making process.^25^ Patients with an active preference for participation may be more active in obtaining information regarding their disease. However, previous studies suggest that even if physicians have the impression that their patients received sufficient information to be able to decide on their treatment, only a small number of patients agreed.^26^ Thus, healthcare professionals should not overestimate patients’ medical knowledge but reconsider to provide more and better information.

Also, in line with previous research, we found that DCP appears to be closely related to patients’ perception regarding their care received. In our analysis, patients with an active DCP were significantly less satisfied with their hospital stay than patients with a passive DCP. Further, we found that patients with an active as well with a collaborative DCP had significantly less trust in the physician and nursing team compared to patients with a passive DCP. A recent study from Thailand, which recruited patients with heart failure, reported similar findings and participants with a collaborative DCP were more dissatisfied with their care compared to patients with a passive DCP.^27^ Also, in a recent study from the USA,^15^ Ruhnke et al. assessed medical inpatients and found that patients who wished to delegate medical decisions to healthcare professionals were more satisfied with their care and had higher trust in the physicians that provided treatment. While these results were based on a mostly African American cohort, in which a large proportion of patients were dependent on Medicaid, our patients came from a more diverse background, suggesting that the association between preference for participation and dissatisfaction is independent from ethnicity and socioeconomic status.

Although we cannot estimate the effect of our findings and the differences in trust and satisfaction appear to be small, we believe our findings are clinically relevant.

There might be different explanations for these associations. Ruhnke et al. suggested that patients with a stronger desire to participate in decision-making might have higher expectations of care and communication or that patients have had previous suboptimal interactions which could possibly impact patients’ DCP and perceived quality of care.^15^ As we found that patients with an active DCP had less trust in their healthcare team, we hypothesize that a lack of trust in the healthcare team might increase patients’ desire to be involved in the decision-making process regarding their own care.

The significant differences in trust in the healthcare team and satisfaction in our study might appear to be small. However, literature has shown that ratings of patient-related experience measures often show ceiling effects with little or no difference.^28^ Still, in our study, we found consistent associations of patients’ DCP with patients’ trust in the healthcare team and satisfaction with hospital stay as two different measures of perceived quality of care, suggesting that our finding is clinically relevant.

Regardless of the mechanism, patient satisfaction is increasingly seen as a critical quality indicator in healthcare and patient-related experience measurements influence hospital reimbursements for care provided.^29–31^ Thus, early identification of active and collaborative patients and use of a more personalized approach may be needed. Consequently, future studies should evaluate whether interventions specifically designed to patients’ DCP may improve patient-reported experience measures.

Further, our results suggest that patients with a passive DCP appear to be less involved in their medical care than patients with a collaborative and active DCP. Ward rounds in patients with a passive DCP were significantly shorter, and sensitive topics were less frequently addressed. At a first glance, this might be in accordance with patients’ preferred role in medical decision-making. However, an Australian trial investigating strategies to adequately respond to patients with different DCPs found that most physicians responded to passive patients by talking most of the time, after outlining their own agendas.^32^ Only few physicians directly addressed patients’ lack of responses, and many did not elicit treatment preferences in passive patients.^32^ Thus, physicians should make sure that these patients are not neglected and that medical decisions taken meet patients’ values and preferences. Regardless of patients’ DCP, literature suggests that actual patient involvement may improve various aspects of quality care such as patient satisfaction^33^ or adherence to treatment regimen^34^ and may decrease healthcare utilization^35^ and charges of malpractice.^36,37^ Interestingly, while our main trial suggested that sensitive topics were less frequently addressed during ward rounds when case presentations were held at the bedside compared to outside the room, in this ancillary analysis, we found that in patients with a passive DCP, sensitive topics were more often addressed at the bedside. This finding suggests that bedside case presentations during ward rounds might be helpful to address personal topics and to facilitate patient-centered care in passive patients.

This study has some limitations. First, this trial took place exclusively in Swiss hospitals, which limits the generalizability of our findings to other countries and cultures. Second, we assessed patients’ knowledge and perceptions regarding the quality of care using a survey developed for this study, which has not been externally validated. Third, as the purpose of the original trial was to assess the effect of bedside and outside-the-room patient case presentation during ward rounds on patient knowledge and other patient-relevant outcomes, we only assessed patients’ preference for participation but not actual participation. Fourth, in our main trial, we did not assess the duration of patients’ hospitalization upon recruitment. However, all participants were only recruited within their first week of hospitalization. Finally, DCP is a self-measure. We may have little knowledge about if patients’ self-declared preference for involvement reflects their real preference and whether patients declaring themselves as “passive” are more satisfied in courses of health care with little involvement than in courses with greater involvement.

## CONCLUSIONS

In conclusion, our results indicate that a patient’s DCP is predictive of his satisfaction with the care provided and trust in the healthcare team. In fact, trust in the healthcare team and overall satisfaction is lower in patients with active DCP despite similar medical knowledge. Moreover, patients with an active DCP had longer ward rounds and sensitive topics were more frequently addressed. Patients with active DCP may need a more personalized approach. Further studies should evaluate whether interventions adapted to patients’ DCP may improve patient-reported experience measures.

## Supplementary Information

ESM 1 (DOCX 216 kb)

## Data Availability

All data will be made available upon request.

## References

[1] O'Hare JA (2008). Anatomy of the ward round. Eur J Intern Med..

[2] **Royal College of Physicians RCoN**. Ward rounds in medicine: principles for best practice. 2015 (London: RCP, 2012.).

[3] O'Mahony S, Mazur E, Charney P, Wang Y, Fine J (2007). Use of multidisciplinary rounds to simultaneously improve quality outcomes, enhance resident education, and shorten length of stay. J Gen Intern Med..

[4] Zwarenstein M, Goldman J, Reeves S (2009). Interprofessional collaboration: effects of practice-based interventions on professional practice and healthcare outcomes. Cochrane Database Syst Rev..

[5] Pucher PH, Aggarwal R, Darzi A (2014). Surgical ward round quality and impact on variable patient outcomes. Ann Surg..

[6] Entwistle VA, Carter SM, Cribb A, McCaffery K (2010). Supporting patient autonomy: the importance of clinician-patient relationships. J Gen Intern Med..

[7] Coulter A, Entwistle V, Gilbert D (1999). Sharing decisions with patients: is the information good enough?. BMJ..

[8] Hamann J, Neuner B, Kasper J, Vodermaier A, Loh A, Deinzer A (2007). Participation preferences of patients with acute and chronic conditions. Health Expect..

[9] Lesley F, Degner JAS (1997). Peri Venkatesh. The Control Preference Scale. Can J Nurs Res..

[10] Degner LF, Kristjanson LJ, Bowman D, Sloan JA, Carriere KC, O'Neil J (1997). Information needs and decisional preferences in women with breast cancer. JAMA..

[11] Hack T, Degner L, Dyck D (1994). Relationship between preferences for decisional control and illness information. Soc Sci Med..

[12] Tricou C, Yennu S, Ruer M, Bruera E, Filbet M (2018). Decisional control preferences of patients with advanced cancer receiving palliative care. Palliat Support Care..

[13] Chiu C, Feuz MA, McMahan RD, Miao Y, Sudore RL (2016). "Doctor, Make My Decisions": Decision Control Preferences, Advance Care Planning, and Satisfaction With Communication Among Diverse Older Adults. J Pain Symptom Manage..

[14] Ross CK, Steward CA, Sinacore JM (1993). The importance of patient preferences in the measurement of health care satisfaction. Med Care..

[15] Ruhnke GW, Tak HJ, Meltzer DO (2020). Association of Preferences for Participation in Decision-making With Care Satisfaction Among Hospitalized Patients. JAMA Netw Open..

[16] Brown RF, Butow PN, Henman M, Dunn SM, Boyle F, Tattersall MH (2002). Responding to the active and passive patient: flexibility is the key. Health Expect..

[17] Becker C, Gamp M, Schuetz P, Beck K, Vincent A, Hochstrasser S (2021). Effect of Bedside Compared With Outside the Room Patient Case Presentation on Patients' Knowledge About Their Medical Care : A Randomized, Controlled, Multicenter Trial. Ann Intern Med..

[18] Schulz KF, Altman DG, Moher D, Group C (2010). CONSORT 2010 Statement: updated guidelines for reporting parallel group randomised trials. Trials..

[19] Charlson ME, Pompei P, Ales KL, MacKenzie CR (1987). A new method of classifying prognostic comorbidity in longitudinal studies: development and validation. J Chronic Dis..

[20] EuroQol G (1990). EuroQol--a new facility for the measurement of health-related quality of life. Health Policy..

[21] Degner LF, Sloan JA, Venkatesh P (1997). The Control Preferences Scale. Can J Nurs Res..

[22] Becker C, Gamp M, Schuetz P, Beck K, Vincent A, Hochstrasser S (2021). Effect of Bedside Compared With Outside the Room Patient Case Presentation on Patients' Knowledge About Their Medical Care : A Randomized, Controlled, Multicenter Trial. Ann Intern Med..

[23] Heesen C, Kasper J, Segal J, Kopke S, Muhlhauser I (2004). Decisional role preferences, risk knowledge and information interests in patients with multiple sclerosis. Mult Scler..

[24] **Deber R, Kraetschmer N, Irvine J**. What Role Do Patients Wish to Play in Treatment Decision Making? 1996. 8678709

[25] Longtin Y, Sax H, Leape LL, Sheridan SE, Donaldson L, Pittet D (2010). Patient participation: current knowledge and applicability to patient safety. Mayo Clin Proc..

[26] Jukic M, Kozina S, Kardum G, Hogg R, Kvolik S (2011). Physicians overestimate patient's knowledge of the process of informed consent: a cross-sectional study. Med Glas (Zenica)..

[27] Pornsiri P, Kanaungnit P, Doungrut W, Chukiat V, Prin V (2019). Predictors of Perceived Quality of Care in People with Heart Failure. Pac Rim Int J Nurs Res..

[28] Badejo MA, Ramtin S, Rossano A, Ring D, Koenig K, Crijns TJ (2022). Does Adjusting for Social Desirability Reduce Ceiling Effects and Increase Variation of Patient-Reported Experience Measures?. J Patient Exp..

[29] Mehta SJ (2015). Patient Satisfaction Reporting and Its Implications for Patient Care. AMA J Ethics..

[30] Lyu H, Wick EC, Housman M, Freischlag JA, Makary MA (2013). Patient satisfaction as a possible indicator of quality surgical care. JAMA Surg..

[31] Cleary PD, McNeil BJ (1988). Patient satisfaction as an indicator of quality care. Inquiry..

[32] Brown RF, Butow PN, Henman M, Dunn SM, Boyle F, Tattersall MHN (2002). Responding to the active and passive patient: flexibility is the key. Health Expect Open Access..

[33] Birkeland S, Bismark M, Barry MJ, Moller S (2022). Is greater patient involvement associated with higher satisfaction? Experimental evidence from a vignette survey. BMJ Qual Saf..

[34] Rachmani R, Levi Z, Slavachevski I, Avin M, Ravid M (2002). Teaching patients to monitor their risk factors retards the progression of vascular complications in high-risk patients with Type 2 diabetes mellitus--a randomized prospective study. Diabet Med..

[35] Bertakis KD, Azari R (2011). Patient-centered care is associated with decreased health care utilization. J Am Board Fam Med..

[36] Finset A (2011). Research on person-centred clinical care. J Eval Clin Pract..

[37] Birkeland S, Bismark M, Barry MJ, Moller S (2021). Does greater patient involvement in healthcare decision-making affect malpractice complaints? A large case vignette survey. PLoS One..

