# Supplementary material for: Patients’ Preference for Participation in Medical Decision-Making: Secondary Analysis of the BEDSIDE-OUTSIDE Trial
Source: J Gen Intern Med. 2022 Sep 9;38(5):1180–9. doi: 10.1007/s11606-022-07775-z (PMC10110786; doi:10.1007/s11606-022-07775-z)
Supplement: Supplementary file 1 — (DOCX 216 kb) [file 11606_2022_7775_MOESM1_ESM.docx]

**Supplementary Appendix**

**eTable 1. Univariable and Multivariable Analyses of Primary and Secondary Outcomes**

| **Outcome measures** | **n** | **Difference or OR (95%C) passive vs SDM** | ***p*** | **Difference or OR (95%C) passive vs active** | ***p*** | **Adjusted difference or OR (95%CI) passive vs SDM  Model 2*** | ***p*** | **Adjusted difference or OR (95%CI)  passive vs active  Model 2*** | ***p*** |
| --- | --- | --- | --- | --- | --- | --- | --- | --- | --- |
| **Primary Endpoint, mean (SD)** |  |  |  |  |  |  |  |  |  |
| Subjective overall knowledge about their medical care (VAS 0-100) | 761 | -2.52 (-6.08, 1.04) | 0.16 | 0.02 (-4.77, 4.80) | 0.99 | -3.21 (-6.76, 0.34) | 0.076 | -0.97 (-5.74, 3.79) | 0.688 |
| **Secondary Endpoints, mean (SD)** |  |  |  |  |  |  |  |  |  |
| Objective overall knowledge about their medical care (rated by study team) | 761 | 2.14 (-2.17, 6.46) | 0.329 | 2.38 (-3.41, 8.18) | 0.42 | 1.34 (-2.91, 5.6) | 0.536 | 1.59 (-4.13, 7.3) | 0.586 |
| **Participation during the ward round, mean (SD) or n (%)** |  |  |  |  |  |  |  |  |  |
| Total duration of ward round per patient (min) | 761 | 0.47 (-0.47, 1.40) | 0.326 | 1.64 (0.39, 2.89) | **0.01** | 0.43 (-0.5, 1.36) | 0.367 | 1.71 (0.46, 2.96) | **0.007** |
| I was encouraged to address personal topics (VAS 0-100) | 550 | -1.91 (-8.26, 4.43) | 0.554 | 8.11 (-0.21, 16.44) | 0.056 | -2.74 (-9.08, 3.6) | 0.396 | 7.43 (-0.84, 15.7) | 0.078 |
| Estimation of my participation during the ward round (VAS 0-100) | 736 | 1.74 (-4.63, 8.11) | 0.592 | 1.22 (-7.31, 9.75) | 0.778 | 1.61 (-4.84, 8.06) | 0.624 | 1.76 (-6.86, 10.39) | 0.688 |
| Time spent with physicians was sufficient (VAS 0-100) | 691 | -2.28 (-5.77, 1.22) | 0.201 | -1.51 (-6.21, 3.18) | 0.527 | -2.09 (-5.59, 1.41) | 0.242 | -0.31 (-5, 4.39) | 0.898 |
| All my questions were answered (VAS 0-100) | 647 | -1.76 (-5.17, 1.66) | 0.313 | 2.45 (-2.02, 6.91) | 0.282 | -2.27 (-5.67, 1.12) | 0.189 | 2.04 (-2.39, 6.47) | 0.366 |
| Occurrence of sensitive topic during the ward round | 760 | 1.15 (0.79, 1.68) | 0.453 | 1.92 (1.11, 3.32) | **0.021** | 1.11 (0.76, 1.62) | 0.604 | 1.84 (1.05, 3.21) | **0.032** |
| **Patient perception regarding discomfort during the ward round** |  |  |  |  |  |  |  |  |  |
| Medical terms used during ward round were confusing (VAS 0-100) | 735 | 1.36 (-3.65, 6.37) | 0.594 | 3.32 (-3.46, 10.1) | 0.337 | 2.22 (-2.73, 7.18) | 0.378 | 3.34 (-3.36, 10.03) | 0.328 |
| I felt discomfort due to the interactions during the ward round (VAS 0-100) | 753 | 2.29 (-0.87, 5.45) | 0.155 | 3.56 (-0.69, 7.81) | 0.101 | 3.71 (-0.7, 8.13) | 0.099 | 4.23 (-1.73, 10.19) | 0.164 |
| Discussion within healthcare team caused upset (VAS 0-100) | 651 | -0.21 (-3.51, 3.09) | 0.899 | 0.84 (-3.71, 5.39) | 0.718 | 0.69 (-2.55, 3.94) | 0.675 | 1.14 (-3.31, 5.6) | 0.614 |
| **Patients' perception regarding quality of care (VAS 0-100)** |  |  |  |  |  |  |  |  |  |
| I felt "in good hands" in this hospital | 756 | -2.15 (-4.78, 0.48) | 0.109 | -3.28 (-6.82, 0.25) | 0.069 | -1.88 (-4.51, 0.75) | 0.162 | -2.72 (-6.26, 0.82) | 0.132 |
| I have trust in the physician team | 751 | -2.95 (-5.61, -0.28) | **0.03** | -5.09 (-8.68, -1.49) | **0.006** | -2.72 (-5.4, -0.04) | **0.047** | -4.74 (-8.35, -1.14) | **0.01** |
| I have trust in the nursing team | 750 | -2.42 (-4.72, -0.13) | **0.038** | -3.41 (-6.5, -0.31) | **0.031** | -2.31 (-4.61, 0) | **0.05** | -2.81 (-5.92, 0.3) | 0.077 |
| There is good collaboration of physicians and nurses | 697 | -1.79 (-4.37, 0.78) | 0.172 | -3.41 (-6.85, 0.04) | 0.052 | -1.56 (-4.11, 0.98) | 0.228 | -2.18 (-5.58, 1.22) | 0.21 |
| I feel physicians have high competence to treat the current illness | 725 | -2.26 (-8.39, 3.88) | 0.471 | 4.01 (-4.22, 12.24) | 0.339 | -1.93 (-8.15, 4.29) | 0.543 | 4.73 (-3.58, 13.03) | 0.264 |
| I feel nurses have high competence to treat the current illness | 729 | -4.26 (-7.94, -0.58) | **0.023** | -4.68 (-9.61, 0.25) | 0.063 | -4.39 (-8.11, -0.68) | **0.021** | -3.99 (-8.96, 0.98) | 0.115 |
| Overall satisfaction with hospital stay | 758 | -3.00 (-5.9, -0.11) | **0.042** | -7.27 (-11.16, -3.38) | **<0.001** | -2.68 (-5.55, 0.19) | 0.067 | -6.23 (-10.08, -2.39) | **0.002** |

**Model 2 adjusted for patient characteristics: age, gender, education*

*Legend: DCP, decisional control preference; OR, Odds ratio; SD, standard deviation; CI, confidence interval, VAS, visual* analog *scale*

**eFigure 1. Patient flow diagram**


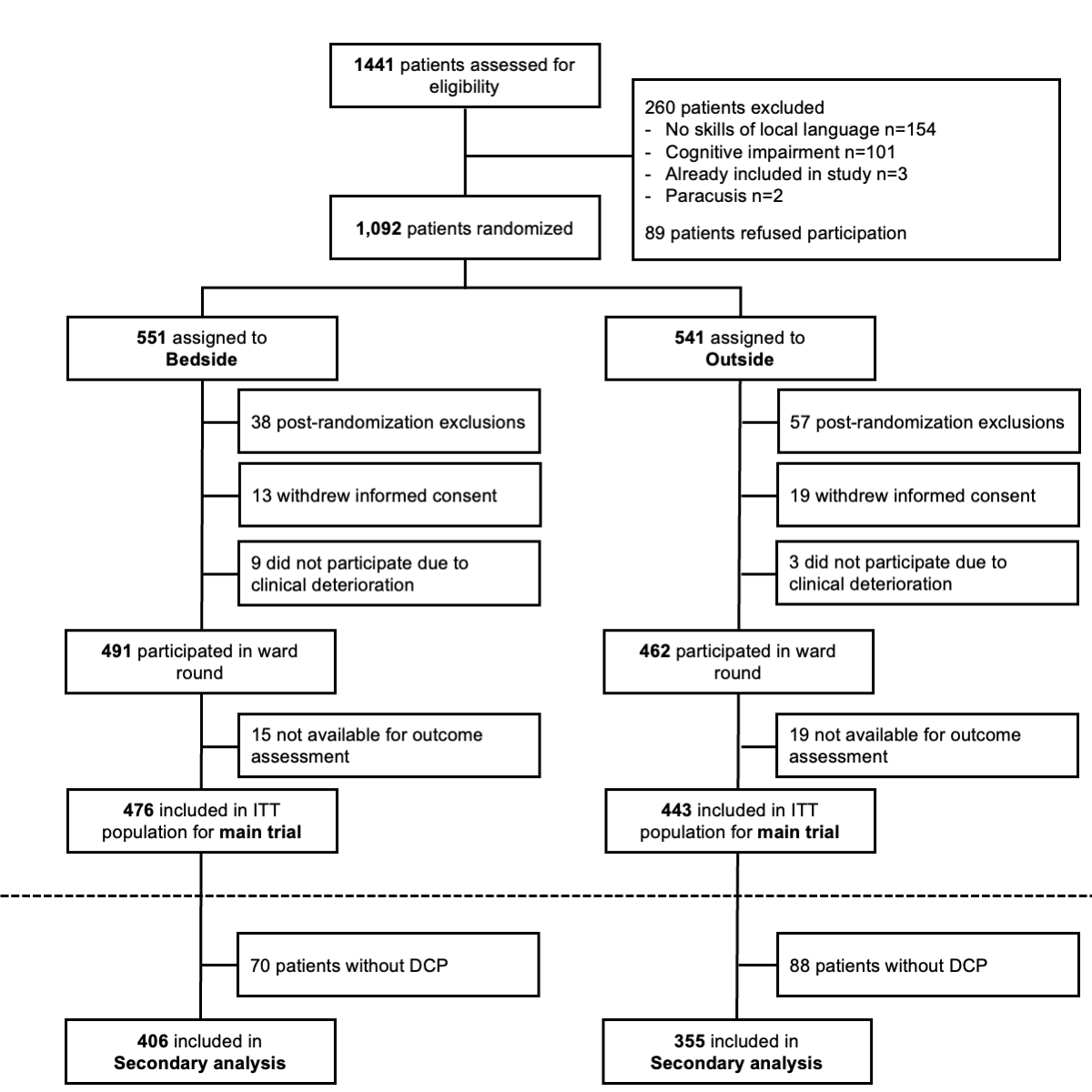


*Legend: ITT, intention to treat; DCP, decisional conflict preference*

**Description of outcomes and its assessment during the trial**

**Primary endpoint** (assessed after the ward round through a structured interview by a blinded member of the research team)

In accordance with our original main trial, the **primary endpoint** of our secondary analysis was defined as patients’ subjective knowledge regarding their medical care (rated on a VAS from 0 to 100: “I have no knowledge at all” to 100 “I have the best possible knowledge”) defined as the average of the visual analogue scale (VAS) score across the following three dimensions:

- 1. Understanding of the disease (“On a scale from 0 to 100, how much do you know about the main disease for your current in-hospital stay?”)
  2. Therapeutic approach (“On a scale from 0 to 100, how much do you know about the therapeutic approach to your current disease?”)
  3. Further plans for care (“On a scale from 0 to 100, how much do you know about further care plans?”)

The primary endpoint was the mean VAS score across these three scores, with a range from 0 to 100 and was assessed after the ward round. We validated the questionnaire for appropriateness and ease of understanding within our physician/researcher team and with patients before initiation of the main trial.

**Secondary endpoints**

**1) Secondary endpoints assessed one day before the ward round**

a) Patients’ preference for participation in medical decision-making.

**2) Secondary endpoints assessed during the ward round**

a) Duration of the ward round per patient (measured by a clock, in minutes)

b) Discussion of sensitive topics during the ward rounds: rated by study team on a nominal 3-point scale (1 = yes; 2 = no; 3 = not applicable).

3) **Secondary Outcomes assessed after the ward round** (through a structured interview by a blinded member of the research team)

a) Patients’ objective knowledge regarding their medical care as rated by the study team.

Analogue to the primary endpoint, objective patients’ knowledge regarding their medical care was defined as the average score across the three individual components, i.e., understanding of the disease, therapeutic approach, and further plans for care, rated on a VAS (0 to 100). Regarding the comprehension of their disease, we categorized the recall of the medical main diagnosis into different categories and rated patients’ responses according to a predefined checklist: (1) patient recalls disease correctly (100 of 100 points), (2) patient recalls either the pathophysiology or localization correctly (e.g., “I have cancer” or “I have a heart condition”, 70 of 100 points), (3) patient recalls symptoms correctly, but does not indicate disease correctly (e.g., “I have shortness of breath” 50 of 100 points) and (4) patient recalls neither symptoms nor disease correctly (0 of 100 points). In case a patient correctly recalled that the disease was still unclear, the response was rated as 100 of 100 points. Regarding therapeutic approach and further plans for care, patients received 70 points each if their response was correct and an additional 30 points if the response was complete. Ultimately, patients’ objective knowledge was defined as the average score of the three individual components. Ratings were performed by two independent raters. Discrepancies between raters were resolved through discussion until consensus was reached.

b) Patients’ perception regarding participation during the ward round.

We evaluated (1) patients’ perception regarding sufficiency of duration of ward rounds, rated on a VAS (0 to 100); (2) patients’ perception of to what degree all their questions were answered rated on a VAS (0 to 100); (3) patients’ estimation regarding their own participation during the ward round rated on a VAS (0 to 100; (4) to what degree patients felt encouraged to address personal issues rated on a VAS (0-100)

c) Patients’ perception regarding discomfort during the ward round, all rated on a VAS (0 to 100): (1) to what degree medical terms used during ward rounds were confusing; (2) to what degree patients felt discomfort due to the interactions during the ward round; (3) to what degree discussion within the healthcare team caused upset.

d) Patients’ perception regarding quality of care: specifically, we focused on the following dimensions, each rated on a VAS (0 to 100): (1) to what degree patients felt they were in “good hands”; (2) trust in nursing and physician team (3) collaboration between physicians and nursing team, (4) perceived competence of physicians and nursing team to treat the current disease as well as overall satisfaction with hospital stay.
